# Polyploidy Expands the Range of *Centaurium* (Gentianaceae)

**DOI:** 10.3389/fpls.2021.650551

**Published:** 2021-03-10

**Authors:** Enrique Maguilla, Marcial Escudero, Vania Jiménez-Lobato, Zoila Díaz-Lifante, Cristina Andrés-Camacho, Juan Arroyo

**Affiliations:** ^1^Departamento de Biología Vegetal y Ecología, Universidad de Sevilla, Seville, Spain; ^2^Laboratorio Nacional de Análisis y Síntesis Ecológica, Escuela Superior de Desarrollo Sustentable, Universidad Autónoma de Guerrero – CONACYT, Chilpancingo de los Bravo, Mexico

**Keywords:** biogeography, *Centaurium*, chromosome, diversification, Mediterranean, niche, polyploidy

## Abstract

The Mediterranean region is one of the most important worldwide hotspots in terms of number of species and endemism, and multiple hypotheses have been proposed to explain how diversification occurred in this area. The contribution of different traits to the diversification process has been evaluated in different groups of plants. In the case of *Centaurium* (Gentianaceae), a genus with a center of diversity placed in the Mediterranean region, polyploidy seems to have been an important driver of diversification as more than half of species are polyploids. Moreover, ploidy levels are strongly geographically structured across the range of the genus, with tetraploids distributed towards more temperate areas in the north and hexaploids in more arid areas towards the south. We hypothesize that the diversification processes and biodiversity patterns in *Centaurium* are explained by the coupled formation of polyploid lineages and the colonization of different areas. A MCC tree from BEAST2 based on three nuclear DNA regions of a total of 26 taxa (full sampling, of 18 species and 8 subspecies) was used to perform ancestral area reconstruction analysis in “BioGeoBEARS.” Chromosome evolution was analyzed in chromEvol and diversification in BAMM to estimate diversification rates. Our results suggest that two major clades diverged early from the common ancestor, one most likely in the western Mediterranean and the other in a widespread area including west and central Asia (but with high uncertainty in the exact composition of this widespread area). Most ancestral lineages in the western clade remained in or around the western Mediterranean, and dispersal to other areas (mainly northward and eastward), occurred at the tips. Contrarily, most ancestral lineages in the widespread clade had larger ancestral areas. Polyploidization events in the western clade occurred at the tips of the phylogeny (with one exception of a polyploidization event in a very shallow node) and were mainly tetraploid, while polyploidization events occurred in the widespread clade were at the tips and in an ancestral node of the phylogeny, and were mainly hexaploid. We show how ancestral diploid lineages remained in the area of origin, whereas recent and ancestral polyploidization could have facilitated colonization and establishment in other areas.

## Introduction

Some areas on Earth have experienced high radiations and become especially rich in the number of species compared to others. These areas of high species richness and elevated percentage of endemism are known as biodiversity hotspots (Myers, 1988). Many factors have been claimed as drivers of increased biodiversity in hotspots, such as long-standing climatic and environmental stability (Fordham et al., 2019), strong spatial variability leading to habitat heterogeneity (e.g., Noroozi et al., 2018), specialized biotic interactions (e.g., Duffy and Johnson, 2017), and prevalence of reproductive systems promoting adaptive novel genetic combinations (Barrett, 2002). One of the drivers which have been proposed is polyploidization, which facilitates both reproductive isolation and adaptation to novel environments (Tate et al., 2005).

Polyploidy has been described to be an important process leading to speciation in plants (Stebbins, 1947; Tate et al., 2005). This mechanism consists of acquiring new sets of chromosomes by hybridization and duplication of the genome (allopolyploidy) or by self-duplication of the whole genome (autopolyploidy). This is a common process; in angiosperms, for example, most species have experienced one or more rounds of polyploidization and subsequent post-polyploid diploidization (Wendel, 2015; Escudero and Wendel, 2020), and at least 47% of species have undergone a recent polyploidy event (Wood et al., 2009). This process can drive speciation promoting reproductive isolation or increasing the probability for new genetic combinations as the species’ genetic content increases. Additionally, polyploids have been demonstrated to tolerate wider climatic and ecological conditions and colonize new areas, facilitated by their increased phenotypic plasticity and higher genetic diversity (see Thompson, 2020).

With about 25,000 plant species, of which about 50% are endemic, the Mediterranean basin region has been considered among the most important biodiversity hotspots (Myers et al., 2000). The Mediterranean climate, rugged topography, heterogeneous geological and geomorphological setting, the isolation provided by many mountains and islands, and historical variations have been considered important causal factors for the huge number of species and fast diversification in the region (Buerki et al., 2012; Rundel et al., 2016). For example, with respect to geography, the formation of the current insular system, especially after the Messinian salinity crisis (5.96–5.33 Ma; Duggen et al., 2003) played a key role on the formation of new species by isolation (Krijgsman et al., 1999; Buerki et al., 2012). Moreover, Anatolia, the Balkans, and the Iberian and Italian peninsulas, have served as refugia for many species during the Quaternary glaciations (Hewitt, 2004; Míguez et al., 2017; Thompson, 2020). Thus, the region became not only an important refuge for many animals and plants but also a source area for the colonization of other regions during the Holocene, after glaciations (Hewitt, 2004; Ali et al., 2019; Thompson, 2020). Climatic oscillations during the Quaternary also led species’ ranges to contract and expand, producing diversification (Avise, 2000; Maguilla et al., 2017; Schneeweiss et al., 2017). Many studies have attempted to understand how diversification takes place in the Mediterranean region using different study cases (e.g., Barres et al., 2013; Maguilla et al., 2017; Affenzeller et al., 2018, etc.). The importance of different traits in the process of diversification depends on the organism under study, and many traits have been proposed to have an impact on speciation or extinction rates (Fitzjohn, 2010).

The genus *Centaurium* Hill (Gentianaceae Juss.), with ca. 26 taxa (18 species and eight subspecies, Mansion and Struwe, 2004; Díaz-Lifante, 2012) has its center of diversity in the Mediterranean basin, although the range of the genus includes Asia, Europe, North-central Africa, and North America (Mansion and Struwe, 2004). In addition to their natural distribution, members of the genus have been introduced into areas of South America, Australia, and New Zealand with similar climates (Mansion and Struwe, 2004). In this genus, 60% of taxa are polyploids (Zeltner, 1970; Mansion et al., 2005), which have been demonstrated to be important in the evolution of *Centaurium* (Mansion et al., 2005). More interestingly, ploidy levels in the genus seem to conform to a geographical pattern, with diploid species (2*n*) close to the Mediterranean basin, tetraploids (4*n*) mainly in northern Europe and eastern Asia, and hexaploids (6*n*) generally distributed in India, the Arabic Peninsula, and southwestern Mediterranean basin reaching the Canary Islands (Mansion et al., 2005). The hexaploids (50% of polyploid taxa) have been proposed to be allopolyploids, while the tetraploids have been suggested to be autopolyploids (Zeltner, 1970; Mansion et al., 2005). However, in many cases, it is very difficult to distinguish allopolyploids from autopolyploids because it is difficult to determine whether gene flow during the course of evolution has occurred between two species or two lineages within the same species. So far, only one clear case of allopolyploidy has been reported for the genus *Centaurium*, in *Centaurium discolor*, in which the two parental species from two different main clades, *Centaurium maritium* and *Centaurium tenuiflorum* formed a new allopolyploid species (Guggisberg et al., 2006).

In this study, we aim to reconstruct the biogeographic history of *Centaurium* as well as its diversification and chromosome evolution in order to understand the geographic pattern of polyploidy and how polyploidization contributed to the evolution, dispersal, and diversification of the genus from its origin in concert with the range expansion of the genus. We hypothesize that the diversification processes and biodiversity patterns in *Centaurium* are explained by the coupled formation of polyploid lineages and the colonization of new areas. To our knowledge, this is a very innovative study with only one recently published study addressing the same macroevolutionary hypothesis but with a different approach (Han et al., 2020).

## Materials and Methods

### Sampling

Samples were collected in the field and from the following herbaria: MA, MGC, NEU, SANT, SEV, and VAL (Thiers, 2015) by Jiménez-Lobato et al. (2019). A Maximum Clade Credibility (MCC) tree constructed by Jiménez-Lobato et al. (2019) using BEAST 2.4.0 (Bouckaert et al., 2014) was used as input for our analyses after removing outgroup species. This MCC tree was based on nuclear DNA regions CPR1, ETS, and ITS (see details in Jiménez-Lobato et al., 2019) and includes all known taxa within genus *Centaurium* following taxonomic treatment by Mansion and Struwe (2004), and Díaz-Lifante (2012) for species occurring in the Iberian Peninsula. In total, 26 taxa (18 species and 8 subspecies) were considered for the genus.

### Ancestral Area Reconstruction

**Figure 1:**
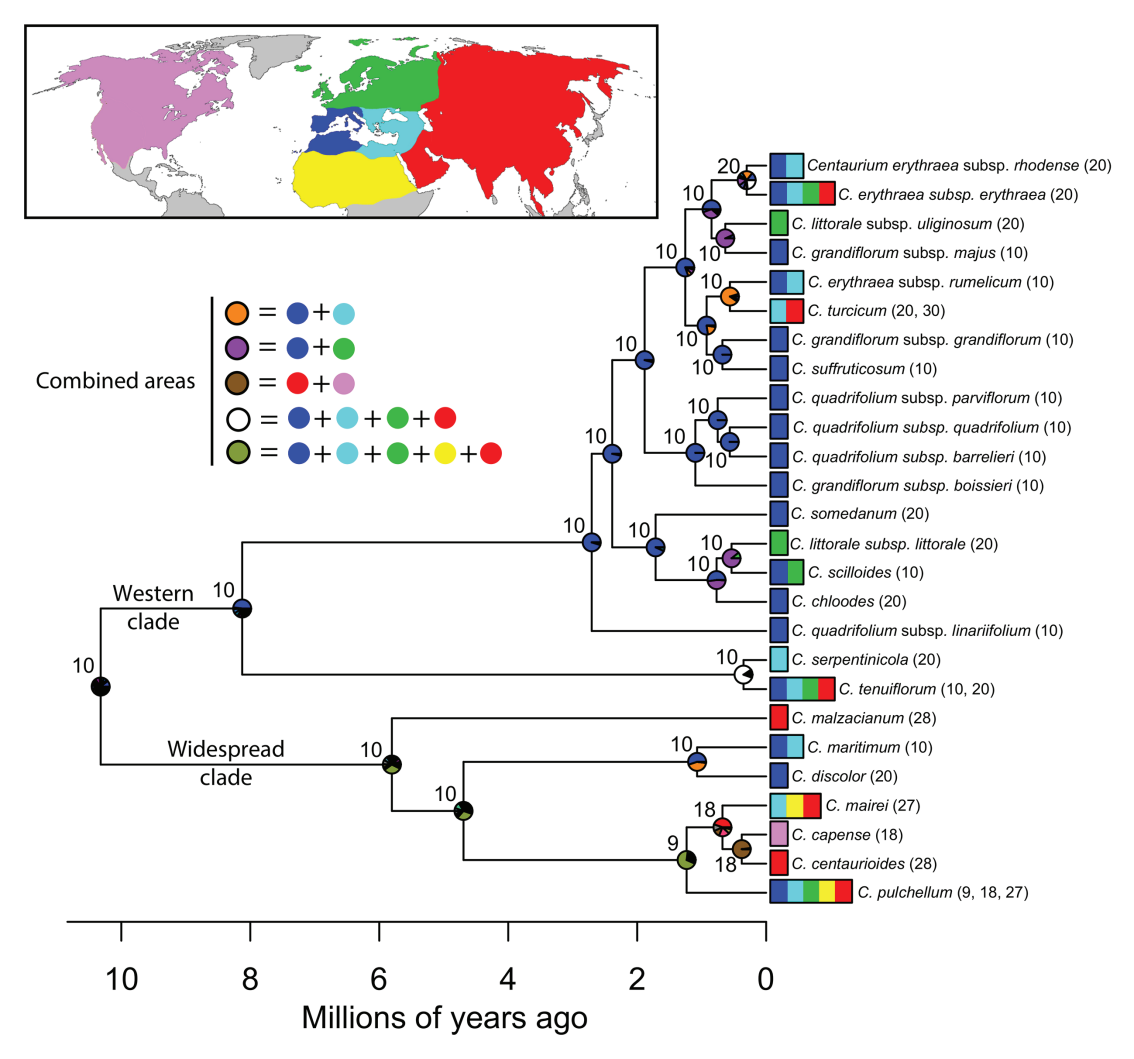
Ancestral area reconstruction under constrained DEC model in *Centaurium* resulting from BioGeoBEARS analysis (Matzke, 2013). Node charts represent the probability of the occurrence of the most recent common ancestor (MRCA) of that node in the area represented by the same color on the map, and combined areas are represented by colors in the legend. Node numbers indicate the reconstructed haploid chromosome numbers retrieved by ChromEvol (Glick and Mayrose, 2014). Tip labels indicate species names and the area where each species occurs following colors in the map. Haploid chromosome number (*n*, in parenthesis) and ploidy levels for the species are also indicated. Lower time-scale shows Millions of years ago, from the origin of the genus to the present.

The package BioGeoBEARS 1.1 (Matzke, 2013) implemented in R 3.2.2 (R Core Team, 2015) was used for the ancestral area reconstruction analyses after coding species’ occurrences within six biogeographic areas (see species scoring in Figure 1): (1) Western Mediterranean, (2) Eastern Mediterranean, (3) northern Europe, (4) northern Africa excluding the Mediterranean region, (5) western and central Asia, and (6) North America. These areas were delimited based on climatic and paleogeographic data following Buerki et al. (2012) with modifications according to species’ distribution patterns in *Centaurium*. The main modification was the split of the Mediterranean into two areas; the western and the eastern Mediterranean, as proposed by other authors (e.g., Takhtajan, 1986) and because many species in the genus are distributed only in the western Mediterranean. Introduced areas were excluded from the scoring of the species’ occurrence matrix.

Three biogeographic models were tested in BioGeoBEARS: Dispersal-extinction-cladogenesis (DEC; Richard and Stephen, 2008), dispersal-vicariance-like (DIVA; Ronquist, 1997), and BayArea-like (Bayesian analysis; Landis et al., 2013). Range expansion and contraction (parameters “*d*” and “*e*”) were allowed to vary during the analyses. Additionally, biogeographic reconstructions were done including founder events or jump dispersal as an additional free parameter (“*j*”). This reached a total of six combinations of models tested. Moreover, all these analyses were repeated including a connectivity matrix based on Hilpold et al. (2014), where dispersal probability between areas was set to one for adjacent areas, 0.5 for areas with an intermediate area between them, and 0.1 for areas separated by more than one area (Supplementary Table 1). In these cases, the parameter “*w*” was considered as a free parameter to reduce subjectivity induced by the assignment of a value for dispersal between geographic areas. The Akaike Information Criterion weight (AICw; Akaike, 1974) was used to compare among the 12 model combinations, selecting the best fit model. As broadly discussed by Ree and Sanmartín (2018), models that include jump dispersal (“*j*”) tend to overestimate cladogenetic dispersal (or jump dispersal) as the main mechanism explaining species’ distribution, considering in many cases null anagenetic rates for the model. For this reason, models including the parameter “*j*” were interpreted with caution and were included mainly to test whether this parameter significantly affected the results. Additionally, to avoid phylogenetic uncertainty bias, we ran in BioGeoBEARS the best fitting model in 100 independent post-burn-in trees from BEAST, and later estimated average probabilities from all trees.

Finally, biogeographic stochastic mapping (BSM; Matzke, 2014) was done in BioGeoBEARS after 50 runs to characterize the number and nature of biogeographical events in the best fitting model. We tested the confidence of the BSM by comparing states probabilities with those obtained under a maximum likelihood (ML) model.

### Chromosome Evolution

The evolution of the chromosome number across the phylogeny was predicted using ChromEvol 2.0 (Mayrose et al., 2010; Glick and Mayrose, 2014). Haploid chromosome numbers (*n*) for all species were obtained from Mansion et al. (2005) and Díaz-Lifante (2012). In the case of species with different ploidy levels, the lowest chromosome number was assumed to be ancestral for the analyses (although only three out of 26 taxa had more than one ploidy level: *Centaurium pulchellum*, *Centaurium turcicum*, and *C. tenuiflorum*). A total of 10 models of chromosome evolution were tested. The analyses were performed following Escudero et al. (2014; but see also Glick and Mayrose, 2014; Aparicio et al., 2019). Akaike’s information criterion (AIC; Akaike, 1974) was used for comparing and choosing the best fitting model of chromosome evolution, which was later used for the reconstruction and plotting of chromosome numbers in the phylogeny (Glick and Mayrose, 2014).

### Diversification Analyses

Diversification rates across the phylogeny were estimated using Bayesian analyses of macroevolutionary mixtures (BAMM; Rabosky et al., 2013, 2014a; Shi and Rabosky, 2015). These analyses were done using BAMM 2.5 in the R package BAMMtools (Rabosky et al., 2014b), allowing changes over time and shifts in diversification rates for five million generations. Markov-Chain Monte Carlo (MCMC) convergence was checked using the R package coda (Plummer et al., 2006), and results of the model with the highest posterior probability (PP) were summarized using BAMMtools.

### Pagel’s Model for Correlated Evolution

Dependent or independent evolution modes of polyploidy and geographic distribution were also tested using Pagel’s method (Pagel, 1994) using the function “fitPagel” from the R package phytools (Revell, 2012). This function searches for correlated evolution of two binary traits. Species and subspecies were coded as diploids against polyploids (including with more than one level of ploidy). Regarding geographic distribution, first, species and subspecies were coded as narrow distributed if they only occur in one area or widespread if they occur in two or more areas. Second, the narrow distributed were coded as exclusively within the area of origin vs. dispersed outside the area of origin, following the biogeographic reconstruction obtained in BioGeoBEARS. For the western clade, the interpretation of the western Mediterranean basin as ancestral range was of high confidence. However, the uncertainty of the ancestral area in the widespread clade was very high. The species with narrow distributions *Centaurium malzacianum*, *C. discolor*, and *Centaurium centauroides* were interpreted as been in their original range as inferred ancestral ranges were widespread ranges including the range of these narrow distributed species. The species *Centaurium capense* from North America was coded as out of the ancestral range based on the reconstruction (as North America was excluded as the possible ancestral distribution in the deeper nodes of the widespread clade). We grouped the widespread species and narrow species out of the ancestral range in one category and the narrow species in the ancestral range in another category. Using these scorings, we tested for the correlation between polyploidy and widespread distribution and/or the expansion to new areas outside of the area of origin of the species or subspecies. We ran models with different rates of transitions between character states (“ARD”), assuming null rates of transition from a polyploid state to a diploid state. Additionally, we performed all analyses using three different models: (i) assuming that the variable polyploidy depends on distribution, (ii) that distribution depends on polyploidy, or (iii) that the two variables depend on each other.

## Results

### Ancestral Areas

The best biogeographic model based on AICc weights (Akaike, 1974) was DEC + Jc (AICc weight = 0.98; Table 1; Supplementary Figure 1); this was a constrained DEC model using the dispersal matrix and setting “*j*” as a free parameter. Nevertheless, given the overestimation of founder events explained above, we interpreted this model with caution. The constrained DEC model (DECc; including dispersal matrix), was the best fitting model with an AICc weight of 0.82 when comparing models without “*j*” as a free parameter (Table 1). In this case, the model showed an extinction rate (“*e*”) of 0.019 events/Myr, and an anagenetic dispersal rate (“*d*”) of 0.085 events/Myr (Table 1). These results were almost identical to the model obtained under the DECc model in BioGeoBEARS in 100 independent post-burn-in trees (see summarize result in Supplementary Figure 2; accordingly, we used the MCC tree results from here).

**Table 1:** Biogeographical models tested in BioGeoBEARS (Matzke, 2013) for *Centaurium*.

Model	LnL	Free params	*d*	*e*	*w*	*j*	AICc	AICcwt	AICcwt without “*j*”
**No constraints**									
DEC	−72.61	2	0.057	2.0e-09	1	0	149.2	0.0022	0.14
DEC+J	−70.39	3	0.051	1.0e-12	1	0.019	164.8	0.0074	–
DIVALIKE	−78.9	2	0.081	0.019	1	0	161.8	4.1e-06	0.0003
DIVALIKE+J	−78.14	3	0.07	0.0046	1	0.016	162.3	3.2e-06	–
BAYAREALIKE	−81.61	2	0.086	0.3	1	0	167.2	2.7e-07	1.8e-05
BAYAREALIKE+J	−77.18	3	0.054	0.15	1	0.034	160.4	8.3e-06	–
**Dispersal multipliers**									
DECc	−69.87	3	0.085	0.019	0.43	0	145.7	0.012	0.82
DEC+Jc	−64.5	4	0.084	1.0e-12	2.07	0.032	137	0.98	–
DIVALIKEc	−72.92	3	0.13	0.021	1.58	0	151.8	0.0006	0.039
DIVALIKE+Jc	−72.08	4	0.11	0.0085	1.54	0.027	152.2	0.0005	–
BAYAREALIKEc	−80.92	3	0.1	0.28	0.12	0	167.8	2.0e-07	1.3e-05
BAYAREALIKE+Jc	−73.25	4	0.09	0.17	1.05	0.062	154.5	0.0002	–

Dispersal-extinction-cladogenesis (DEC), dispersal-vicariance like (DIVA), and Bayesian binary-like (BAYAREALIKE) models were tested with and without the “*j*” parameter which allows founder speciation events (cladogenetic dispersal rate). Additionally, all analyses were performed adding a dispersal multiplier matrix where dispersal probability was set to 1 for adjacent areas, 0.5 for areas with an intermediate area between them, and 0.1 for areas separated by more than one area. In these cases, “*w*” was considered as a free parameter to reduce subjectivity induced by the assignment of a given value for dispersal between geographic areas. LnL indicates Log-likelihood for each model tested, numbers of free parameters included as “Free Params,” “*d*” for anagenetic dispersal rate, “*e*” for extinction, Akaike’s information criterion corrected for small sample size (AICc), Akaike’s information criterion weights for all eight models (AICcwt) and considering only models without “*j*” parameter. The best fitted model without “*j*” is highlighted in bold type.

**Figure 2:**
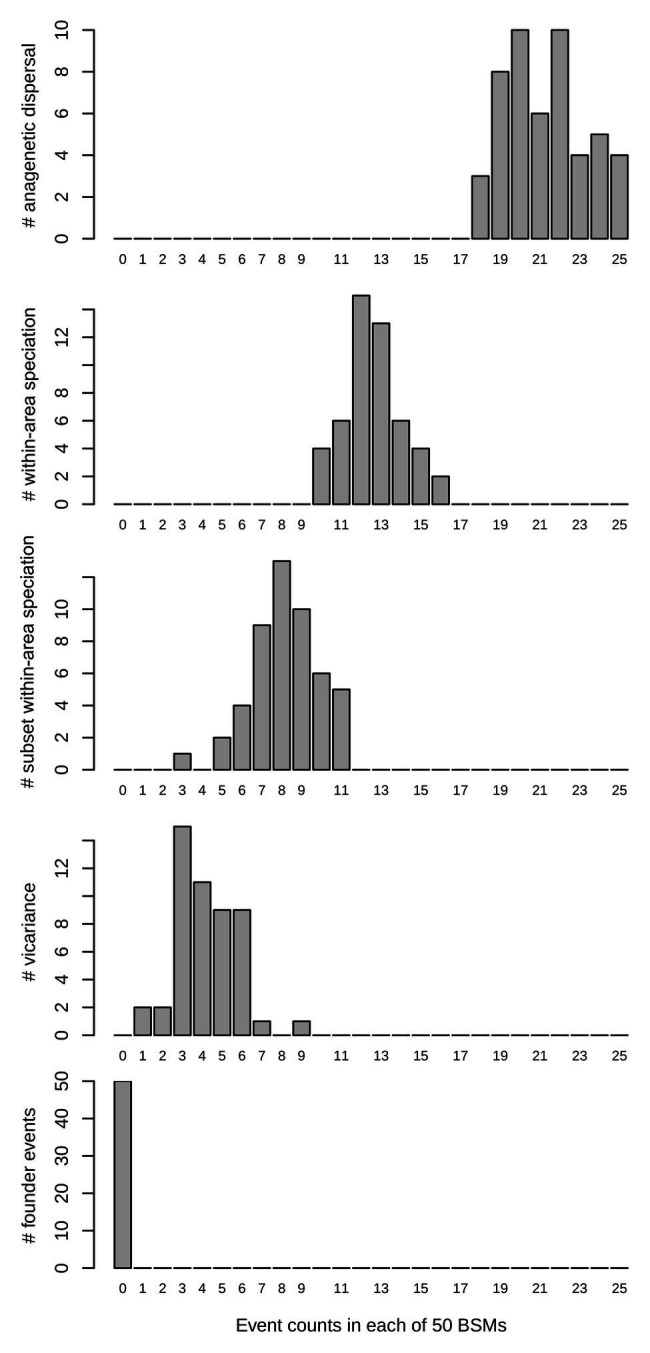
Biogeographical events count after 50 biogeographic stochastic mappings (BSMs) in the best fitting model obtained in BioGeoBEARS (constrained DEC model) in *Centaurium*. The horizontal (*x*) axis represents the number of events, and the vertical (*y*) axis the number of BSM analyses.

**Figure 3:**
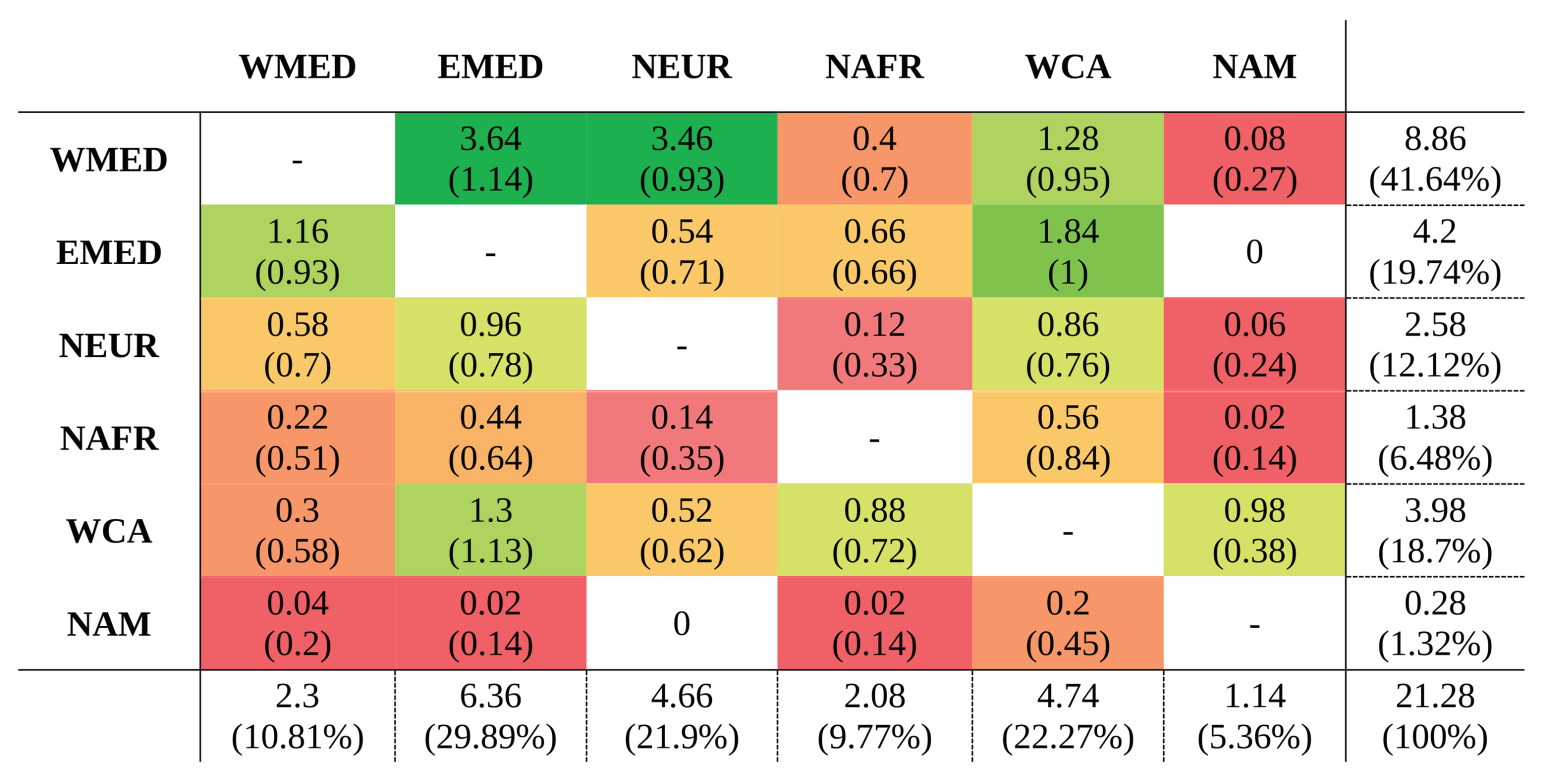
Average number of dispersals and standard deviation (in parenthesis) after 50 BSMs obtained from BioGeoBEARS (Matzke, 2013) under the constrained DEC model in *Centaurium*. Source and sink areas are indicated in the *y* and *x* axes respectively, codified as follows: WMED for western Mediterranean; EMED, eastern Mediterranean; NEUR, northern Europe; NAFR, northern Africa; WCA, west and central Asia; and NAM for North America. The frequency of each event is also indicated by the cell color (green colors for more frequent events, and warmer colors for infrequent or null events). The last row and column summarize the dispersal events or destination of each area, respectively, indicating also the percentage of all events.

After the most recent common ancestor (MRCA) of the whole genus *Centaurium* around 10 million years ago (Ma), this phylogeny showed an early split into two major clades (Figure 1). For the first major clade (hereafter the western clade) comprising most species and subspecies from the western Mediterranean and some species and subspecies from surrounding areas (the rest of the Palaearctic), the MRCA was most likely predicted to be from the western Mediterranean (Figure 1; other possible ancestral ranges were unlikely and included western Mediterranean as part of the ancestral distribution). The origin of the MRCA for the second major clade (hereafter the widespread clade) was unresolved, as occurred with other internal nodes within this clade, suggesting wider ancestral areas. This second major clade was composed of *C. capense*, *C. centaurioides*, *C. discolor*, *Centaurium mairei*, *C. malzacianum*, *Centaurium maritimum* and the broadest distributed species in the genus, *C. pulchellum*, which is present in all codified areas (but North America). In the evolution of *Centaurium*, anagenetic dispersal was apparently the most important mechanism explaining the current distribution and biogeographic history of the genus (a mean of 21.28 ± 2.02 events after 50 BSMs; Figure 2). The area of origin of most *Centaurium* species and subspecies, the western Mediterranean, acted as a source for many dispersals to other areas (41.64% of all dispersal events; Figures 1, 3). Most of these dispersals took place from the western Mediterranean to the eastern Mediterranean and northern Europe (a mean of 3.64 and 3.46 events, respectively; Figure 3), and most of them took place towards the tips of the phylogeny (Figure 1). This mechanism was followed in importance by within-area speciation, which showed a mean of 12.62 ± 1.48 events after 50 BSM runs. Less important were subset within-area speciation (a mean of 8.18 ± 1.72 events) and vicariance (4.2 ± 1.55 events; Figure 2). Finally, a comparison of state probabilities obtained using Bayesian stochastic mapping and ML approaches were compared to test the suitability of the model, obtaining an *R^2^* of 0.985 (Supplementary Figure 3), which confirmed that states predicted after the 50 BSMs were strongly congruent with a ML approach.

### Diversification Rates

**Figure 4:**
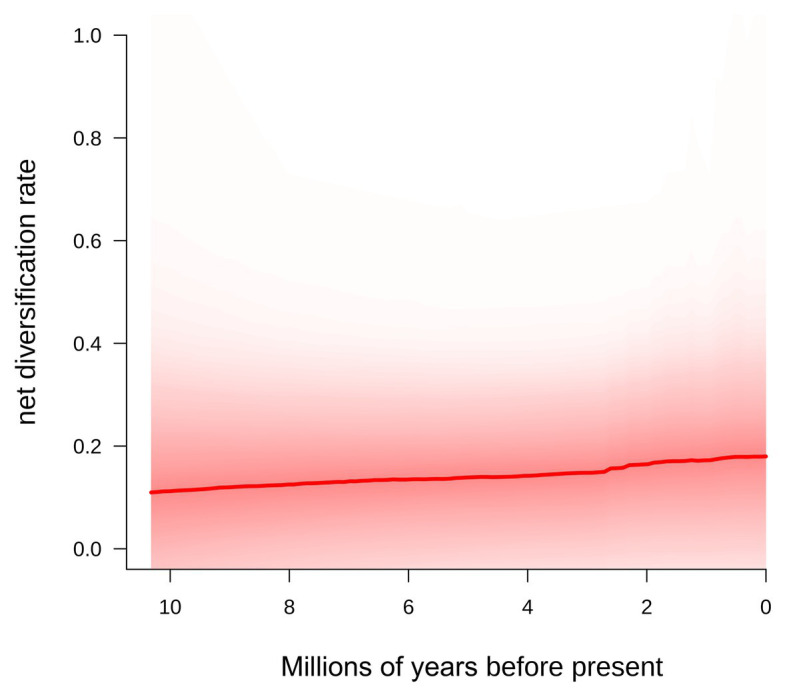
Diversification rate from the origin of the genus *Centaurium* to the present obtained using BAMM (Rabosky et al., 2013, 2014a; Shi and Rabosky, 2015).

The BAMM analyses showed that diversification rates were slightly increasing towards the present in *Centaurium* (Figure 4). A scenario without shifts in diversification rates was the most plausible (probability = 0.811), followed by a scenario with one shift in diversification rates, though this scenario had a probability of only 0.137. The speciation rate for the most supported model (0-shifts) was 0.654 lineage per million years (lin./Myr; lam1), with an extinction rate of 0.546 lin./Myr (mu1) and speciation growth parameter of 0.018 lin./Myr (lam2; Figure 4).

### Chromosomal Evolution and Biogeography

The best-fitting model for chromosome evolution based on AIC values was “BASE_NUM_DUPL” (AIC = 116.989; see Supplementary Table 2 for AIC values of all models tested), which considered that chromosome changes were driven by the gain or loss of a chromosome pair, whole-genome duplication (polyploidization) or changes in the basic number (Glick and Mayrose, 2014). This scenario of chromosome evolution showed a rate of 0.52 loss events/Myr, and a gain of 0.00 events/Myr. The rate for duplication of the chromosome number was 0.81 duplications/Myr. For the MRCA of *Centaurium*, the estimated chromosome number was *n* = 10 (Figure 1). This is expected to be the ancestral chromosome number for the genus. Our results showed how polyploidization occurred mainly in the tips of the phylogeny in the case of the large western clade (only one polyploidization event inferred in the very shallow node of the subspecies *Centaurium erythraea* ssp. *erythraea* and ssp. *rhodense*), whereas in the widespread clade, there is one ancestral polyploidization event reconstructed at the MRCA node of the species *C. mairei*, *C. capense*, and *C. centauroides*. Specifically, a chromosome loss event was predicted for the MRCA of the clade composed of *C. capense*, *C. centaurioides*, *C. mairei*, and *C. pulchellum* (base number shifts from 10 to 9), and a later polyploidization event in the case of the MRCA of *C. capense*, *C. centaurioides*, and *C. mairei*.

**Figure 5:**
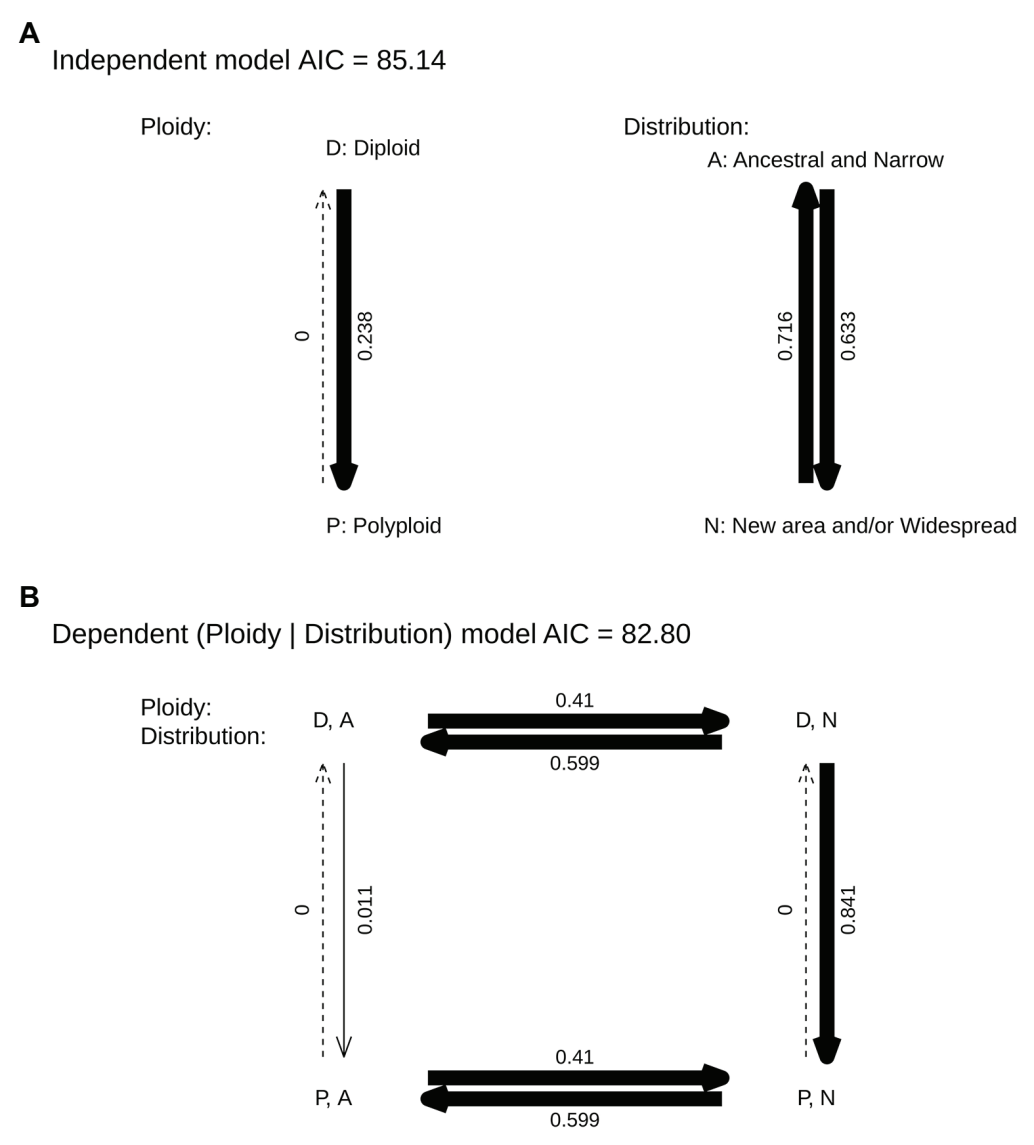
Pagel’s binary correlation test of polyploidy (D for diploid, P for polyploid) and the presence in the area of origin of the species (A if the species is in the ancestral area, N if the species has colonized a new area even if it is still present in its ancestral area and/or have widespread distribution) in *Centaurium*. Independent (A) and dependent (B) models are shown. Arrow thickness indicates rate proportion.

The analyses using Pagel’s method retrieved dependent correlated evolution between polyploidy and geography, where polyploidy depended on the area where the species and subspecies occurs (Figure 5). Initially, the dispersal rate was independent of polyploidy (from the area of origin to new areas 0.410 events/Myr and *vice-versa*, 0.599 events/Myr). Second, whereas transitions from diploidy to polyploidy were almost null in the area of origin (0.011 events/Myr), the rate of transition from diploidy to polyploidy in species and subspecies inhabiting new areas or with widespread distribution was 0.841 events/Myr.

## Discussion

### Origin and Evolution of *Centaurium*

Based on the best fitting model (DECc, AICw = 0.82; Table 1), two main clades – the western and widespread clade – originated from their MRCA. Nevertheless, the best fitting model (regardless of whether founder event “*j*” was included as a free parameter) could not confidently reconstruct the origin of the MRCA of the whole genus *Centaurium* (Table 1; Figure 1; Supplementary Figure 1), originated during the Miocene (Jiménez-Lobato et al., 2019). The western clade that originated most likely in the western Mediterranean, which includes almost 70% of extant taxa in the genus (Figure 1), started diversifying about 8.1 Ma (Jiménez-Lobato et al., 2019). Most taxa in this clade occur in the western Mediterranean, only one species with two subspecies (*Centaurium littorale*) is not present in the Mediterranean but only in northern Europe. The other clade (with ca. 30% of extant taxa) has up to 40% of the species and subspecies with widespread ranges and combining different distribution patterns including the six coding areas in our analyses. For example, *C. pulchellum* is present in all of the codified areas (but North America), *C. mairei* in the eastern Mediterranean, Africa, and west and central Asia, and *C. maritimum* in the whole Mediterranean region, whereas other species have more restricted distribution; for example, *C. capense* is found in North America and *C. centaurioides* and *C. malzacianum* in west and central Asia, and *C. discolor* in the western Mediterranean (Figure 1). This complex and wide range of species occurrences made BioGeoBEARS unable to reconstruct the geographic origin of deeper nodes in this clade, leaving the biogeographic history of these species partially unknown. The ancestral distribution of the widespread clade was inferred as a broad distribution including west and central Asia but the specific composition of this inferred broad ancestral distribution is very uncertain.

In *Centaurium*, as in many other groups of plants, the Mediterranean basin acted as a source for later colonization of adjacent areas after glaciations (Thompson, 2020). The origin of most dispersal events in the genus was the western Mediterranean (Figure 3). The most common destinations were adjacent areas, such as the eastern Mediterranean and northern Europe when the origin was the western Mediterranean (with a mean of 3.64 and 3.46 dispersal events, respectively; Figure 3), and the western Mediterranean and west and central Asia in the case of dispersal from the eastern Mediterranean (a mean of 1.16 and 1.84 events, respectively; Figure 3). Interestingly, most dispersal events occurred in recent times in the phylogeny, and all species from the Mediterranean dispersed northward and eastward. Thus, the colonization of southern latitudes, i.e., northern Africa, occurred mainly from western and central Asia and not from the western Mediterranean (Figure 3). Regarding the colonization of North America, the MRCA of *C. capense* and *C. centaurioides* presumably dispersed from Asia to North America over the Bering Land Bridge (BLB), when this was open during glacial periods and later diversified into two species: *C. capense* from North America, and *C. centaurioides* in west and central Asia, probably during the past million years (Figures 1, 3). The BLB connected Asia and North America during periods where the sea level descended as a consequence of ice formation during glacial periods (Hopkins, 1972; Brikiatis, 2014). The formation of the BLB occurred alternatively during glacial and interglacial periods since the Pliocene (Harris, 2005) and was used by many plant species as a route of dispersal from one continent to the other (e.g., Harris, 2007; Waltari et al., 2007; Mao et al., 2010; Maguilla et al., 2018; among others). The crown node of the separation between *C. capense* and *C. centaurioides* was dated by Jiménez-Lobato et al. (2019) sometime during the Pleistocene. This coincides with a period where the BLB was successively closed and then Asia and North America separated (Harris, 2007; Figure 2), supporting the role of the Bering Strait and, more particularly, the alternate opening and closure of the BLB as a dispersal and speciation driver in this clade (Figure 1).

Thus, the importance of the Mediterranean basin for *Centaurium* lies not only in the fact that it is the place of origin of the MRCA of 70% of the genus (Figure 1), but also as a region where lineages diversified, showing a constant increase in diversification rates from the origin to the present (Figure 4). Presumably, the role of some parts of the Mediterranean as glacial refugia (Petit et al., 2003) allowed diversification of the genus in the region, and were the origins for later dispersals, mainly to adjacent areas during interglacial periods, where more favorable niches were available in upper latitudes.

### Polyploidy as a Key to Expand the Range

Diversification in *Centaurium* was dominated by multiple events of anagenetic dispersals, followed by within-area speciation, then less frequent subset within-area speciation (this is, speciation within part of the distribution of the ancestor but not over the whole area) and rarely, vicariance, as commented above (Figure 2). According to biogeographic reconstruction (Figure 1), most within-area speciation occurred in the western Mediterranean, where most of the species and subspecies in the genus originated. Later, lineages diversified at a constantly increasing rate from the origin to the tips of the phylogeny (Figure 4), while at the same time dispersing, mainly into adjacent areas (Figure 3). When a plant species disperses or colonizes a new area or niche, the success of the event depends on multiple traits. High phenotypic plasticity, capacity for sexual reproduction, high competitiveness with other species or wide range of climatic tolerance are only a few examples of factors affecting the success of establishment in a new area (Baker, 1965; Levin, 2000; Maguilla et al., under review). Polyploidization has been described to have important evolutionary consequences and correlate strongly with range expansion in plants (Levin, 1983; Hijmans et al., 2007; Treier et al., 2009; Te Beest et al., 2012; Soltis et al., 2015). In the Mediterranean region, polyploidy is common among plants, and polyploidization has been demonstrated to be an important trait for diversification in species’ lineages within the region (Vilatersana et al., 2000; Escudero et al., 2018; Thompson, 2020), although it can either increase or decrease rates of diversification (Escudero et al., 2018).

Cytogenetic studies reported two basic chromosome numbers for *Centaurium* (*x* = 9 and *x* = 10; Zeltner, 1970) and during the evolutionary history of the genus, chromosome numbers remained invariable at deeper nodes from an ancestral chromosome number of *n* = 10 (Figure 1). In the western clade, with most species and subspecies occurring in the area of origin, polyploidization events took place at the tips (with only one exception in a very shallow clade that contains two subspecies), whereas in the widespread clade, with up to ca. 40% of species broadly distributed and inferred broad ancestral areas, a polyploidization event has been reconstructed to be older, as reflected in Figure 1. It is interesting that the widespread clade where up to 40% of species are broadly distributed is also the clade where the percentage of polyploidization events is highest and events deeper in our chromosome number reconstruction (Figure 1). Whereas in the western clade the percentage of polyploids is ca. 50% (mostly tetraploids), the percentage of polyploids in the widespread clade rises to 70% (mostly hexaploids that curiously mostly avoid the Mediterranean region with a clear preference for more arid regions). This widespread clade includes for example *C. pulchellum*, with a widespread distribution across the northern hemisphere, occurring in all five areas codified for our analyses but North America (Figure 1). Except for *C. maritimum* from the western and eastern Mediterranean, all other species in this clade (i.e., *C. capense*, *C. centaurioides*, *C. discolor*, *C. mairei*, *C. malzacianum*, and *C. pulchellum*) show some level of polyploidization (Figure 1).

When analyzing the possible correlation between polyploidy and distribution, we found a strong dependence of polyploidy on distribution (Figure 5). Thus, our results confirmed that polyploidy occurred in species and subspecies that are widespread or dispersed out of the area of origin, and suggest that it may have facilitated their establishment and success in the newly colonized area. Thus, polyploidization does not seem to facilitate the dispersal event *per se*, but rather success in the new or expanded area (Figure 5). Additionally, Mansion et al. (2005) represented geographically the ploidy level of *Centaurium* species in the Mediterranean basin showing that tetraploids dominated upper latitudes, whereas hexaploids are more common in southern latitudes and diploids in the core of the Mediterranean basin. This is congruent with polyploidy favoring adaptation to different niches or new areas.

## Conclusion

We demonstrated how *Centaurium* species in the western clade remaining in their ancestral area, the western Mediterranean, are diploids. Both recent and ancestral polyploidization events have facilitated the expansion of the distribution range and/or the colonization of new areas outside the areas of origin. Interestingly, *Centaurium* tetraploid species were more successful colonizing north of the Mediterranean in a more temperate climatic regime. Hexaploid species seem to be more successful growing in southern places in a more arid climatic regime (although our results are not conclusive on whether or not the current range of the hexaploid species is ancestral or recently colonized).

## Data Availability Statement

The original contributions presented in the study are included in the article/Supplementary Material, further inquiries can be directed to the corresponding author.

## Author Contributions

JA and ME conceived the research topic. CA-C, VJ-L, and ZD-L gathered chromosome data and helped assessing plant taxonomy and distributions. EM and ME performed the analyses. EM wrote the manuscript. All authors contributed to the article and approved the submitted version.

### Conflict of Interest

The authors declare that the research was conducted in the absence of any commercial or financial relationships that could be construed as a potential conflict of interest.
