# Supplementary material for: Polyploidy Expands the Range of Centaurium (Gentianaceae)
Source: Front Plant Sci. 2021 Mar 10;12:650551. doi: 10.3389/fpls.2021.650551 (PMC7988210; doi:10.3389/fpls.2021.650551)
Supplement: Supplementary file 1 [file Data_Sheet_1.docx]

Supplementary Material

**
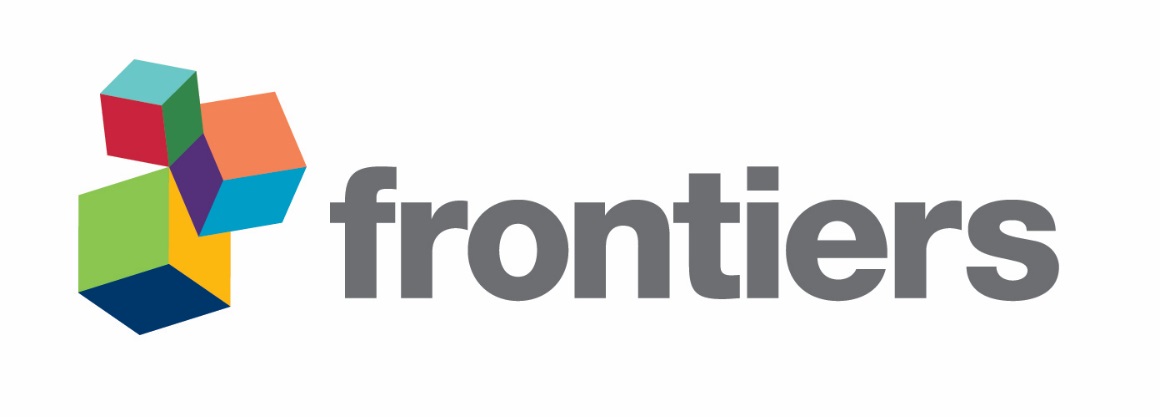
**

**Supplementary Figure 1.**


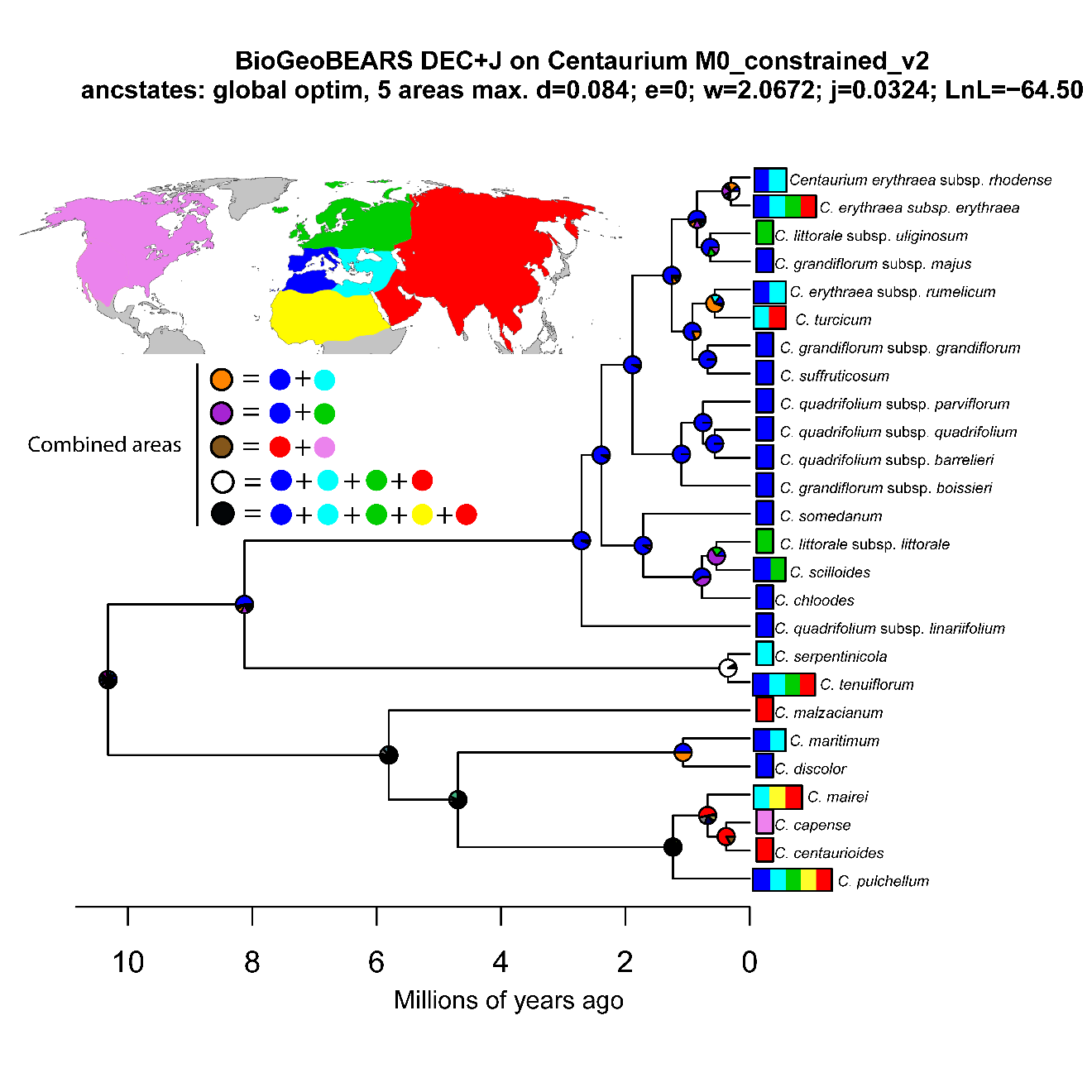


**Supplementary Figure 1.** Ancestral area reconstruction under constrained DEC model and free parameter “j” which allows jump dispersal in *Centaurium,* resulting from BioGeoBEARS analysis (Matzke, 2013). Node charts represent the probability of the occurrence of the most recent common ancestor (MRCA) of that node in the area represented by the same color on the map, and combined areas are represented by colors in the legend. Tip labels indicate species names and the area where each species occurs following colors in the map. Lower time-scale shows Millions of years ago, from the origin of the genus to the present.

**Supplementary Figure 2.**


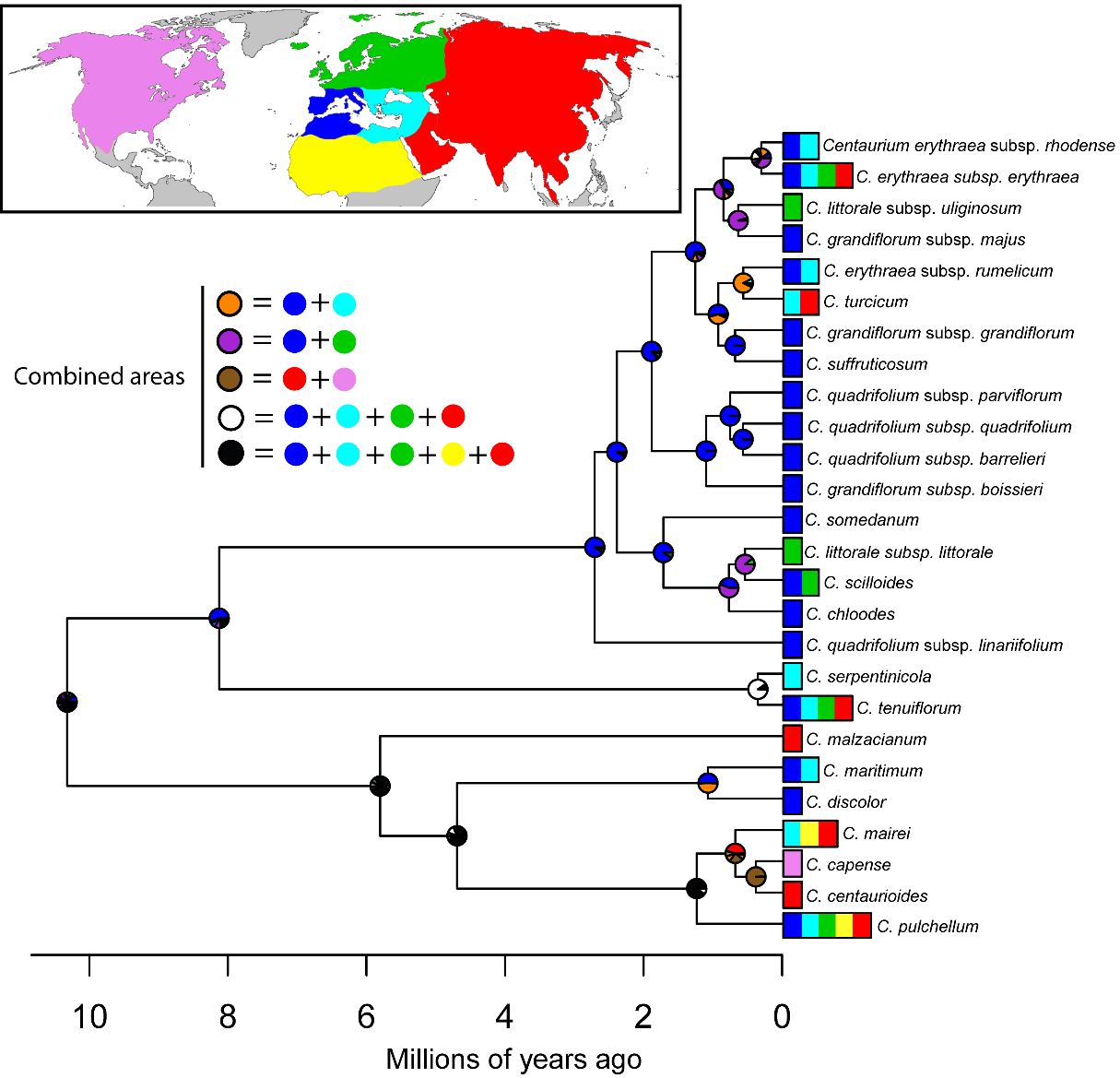


**Supplementary Figure 2.** Ancestral area reconstruction under constrained DEC model in *Centaurium,* showing mean results from independent ancestral area reconstructions in the “BioGeoBEARS” (Matzke, 2013), using 100 post-burn-in maximum clade credibility trees from BEAST. Node charts represent the probability of the occurrence of the most recent common ancestor (MRCA) of that node in the area represented by the same color on the map, and combined areas are represented by colors in the legend. Tip labels indicate species names and the area where each species occurs following colors in the map. Lower time-scale shows Millions of years ago, from the origin of the genus to the present.

**Supplementary Figure 3.**





**Supplementary Figure 3.** Comparison of state probabilities under constrained DEC model in *Centaurium* (Gentianaceae), obtained using Bayesian stochastic mapping and maximum likelihood approaches.

**Supplementary Table 1**. Connectivity matrices between geographic realms (1 – Western Mediterranean, 2 – Eastern Mediterranean, 3 – Northern Europe, 4 – Northern (non-Mediterranean) Africa, 5 –West and Central Asia, 6 – North America) used as input for “BioGeoBEARS” analyses including dispersal multipliers based on Hilpold et al. (2014). This is, 1 for adjacent areas, 0.5 for areas with an intermediate realm between them, and 0.1 for areas separated by more than one realm.

|  | **1** | **2** | **3** | **4** | **5** | **6** |
| --- | --- | --- | --- | --- | --- | --- |
| **1** | 1 | 1 | 1 | 1 | 0.5 | 0.1 |
| **2** | 1 | 1 | 1 | 1 | 1 | 0.1 |
| **3** | 1 | 1 | 1 | 0.5 | 1 | 0.1 |
| **4** | 1 | 1 | 0.5 | 1 | 1 | 0.1 |
| **5** | 0.5 | 1 | 1 | 1 | 1 | 0.5 |
| **6** | 0.1 | 0.1 | 0.1 | 0.1 | 0.5 | 1 |

**Supplementary Table 2.** Akaike’s information criterion (AIC) values of all models of chromosome evolution tested in ChromEvol for *Centaurium*. Best fitting model is shown in bold letter.

| **Model** | **Log Likelihood** | **AIC** |
| --- | --- | --- |
| BASE_NUM | -56.2311 | 120.462 |
| **BASE_NUM_DUPL** | **-53.4947** | **116.989** |
| CONST_RATE | -82.6371 | 171.274 |
| CONST_RATE_DEMI | -57.9445 | 121.889 |
| CONST_RATE_DEMI_EST | -56.5147 | 121.029 |
| CONST_RATE_NO_DUPL | -98.4156 | 200.831 |
| LINEAR_RATE | -81.0634 | 172.127 |
| LINEAR_RATE_DEMI | -57.5882 | 125.176 |
| LINEAR_RATE_DEMI_EST | -55.2665 | 122.533 |
| LINEAR_RATE_NO_DUPL | -91.7715 | 191.543 |
